# Meteorological Report for September

**Published:** 1880-10

**Authors:** 


### METEOROLOGICAL REPORT FOR SEPTEMBER, 1880.

| RAIN. DATE | RAIN. IN. | Mean daily Barometer. | Mean daily Thermometer | Mean daily Humidity. | Maximum Thermometer | Minimum Thermometer | Prevailing direction of Wind. | Maximum hourly velocity of Wind, (Daily.) |
|---|---|---|---|---|---|---|---|---|
| 1 | 14 | 20.930 | 74.7 | 83.3 | 85 | 71 | S. E. | 24 |
| 2 | 05 | 29.993 | 76.0 | 84.0 | 83 | 70 | S. E. | 16 |
| 3 | 0 | 29.962 | 77.7 | 76.0 | 84 | 70 | N. E. | 12 |
| 4 | 0 | 29.946 | 81.0 | 66.0 | 86 | 71 | S. | 9 |
| 5 | 25 | 30.010 | 77.2 | 74.0 | 86 | 71 | S. W. | 8 |
| 6 | 03 | 29 940 | 78.2 | 71.7 | 87 | 71 | E. | 16 |
| 7 | 32 | 29.825 | 75.0 | 77.7 | 83 | 69 | N. W. | 23 |
| 8 | 32 | 29.862 | 65.2 | 87.3 | 71 | 62 | N. E. | 14 |
| 9 | 0 | 30.045 | 62.5 | 68.0 | 70 | 59 | N. W. | 16 |
| 10 | 0 | 30.219 | 66.0 | 77.7 | 72 | 53 | N. E. | 16 |
| 11 | 37 | 30.270 | 59.5 | 94.0 | 67 | 58 | E. | 21 |
| 12 | 18 | 30.172 | 67.2 | 87.7 | 74 | 57 | E. | 8 |
| 13 | 1.01 | 29.986 | 67.0 | 87.3 | 70 | 64 | N. W. | 13 |
| 14 | 0 | 30.012 | 63.2 | 64.7 | 70 | 55 | N. W. | 13 |
| 15 | 0 | 30.066 | 62.0 | 67.0 | 69 | 54 | N. E. | 11 |
| 16 | 0 | 30.062 | 67.0 | 63.7 | 75 | 55 | N. | 9 |
| 17 | 0 | 30.106 | 70.2 | 62 0 | 78 | 60 | N. W | 9 |
| 18 | 0 | 30.031 | 72.0 | 59.3 | 80 | 60 | E. | 7 |
| 19 | 0 | 30.140 | 74.5 | 59.0 | 81 | 65 | S. E. | 10 |
| 20 | 0 | 30.121 | 75.5 | 64.7 | 82 | 65 | S. E. | 9 |
| 21 | 0 | 30 087 | 71.7 | 67 3 | 82 | 64 | N. W, | 13 |
| 22 | 0 | 30.092 | 70.7 | 69.7 | 81 | 59 | N. | 9 |
| 23 | 0 | 30.103 | 71.2 | 68.0 | 81 | 62 | E. | 16 |
| 24 | 3.17 | 30.145 | 64.5 | 94 0 | 70 | 60 | E. | 18 |
| 25 | 13 | 30 080 | 66.5 | 90.7 | 72 | 63 | E. | 12 |
| 26 | 0 | 30.008 | 72.0 | 81.3 | 78 | 65 | S. E. | 12 |
| 27 | 15 | 29.856 | 72.5 | 82.3 | 82 | 66 | S. E. | 20 |
| 28 | 0 | 29.974 | 61.5 | 67.0 | 67 | 59 | N. W. | 20 |
| 29 | 0 | 30.087 | 61.7 | 57 3 | 73 | 54 | N. W. | 21 |
| 30 | 0 | 30.202 | 58.5 | 56.3 | 66 | 47 | N. W. | 12 |
| | | ........... | ...... | ...... | ... | ... | ......... | ... |
| Sums | | 30 050 | 69.4 | 73.3 | 76.8 | 62.5 | ......... | ... |

Highest Barometer, 30.291, on the 11th.
Lowest Barometer, 29.772, on the 7th.
Monthly range of Barometer, 0.519.
Highest Temp. 87, on 6th. Lowest Temp. 47°, on 30th.
Monthly range of Temperature, 40°.
Greatest daily range of Temperature, 22°, on the 22d.
Least daily range of Temperature, 6°, on the 13th.
Mean of Maximum Temperatures, 76°.8.
Mean of Minimum Temperatures, 62°.0.
Total rainfall or melted snow, 6.21 inches.
Prevailing wind, N. W. Total movement of wind, 54.08 miles.
Maximum velocity of wind and direction, 24 miles per hour; S.E., on 1st.
Total number of days on which rain fell, 12.

H. HALL, Corp'l. Signal Service U. S. A

Atlanta, Ga., September, 1880.

